# Bats Respond to Very Weak Magnetic Fields

**DOI:** 10.1371/journal.pone.0123205

**Published:** 2015-04-29

**Authors:** Lan-Xiang Tian, Yong-Xin Pan, Walter Metzner, Jin-Shuo Zhang, Bing-Fang Zhang

**Affiliations:** 1 Biogeomagnetism Group, PGL, Key Laboratory of Earth and Planetary Physics, Institute of Geology and Geophysics, Chinese Academy of Sciences, Beijing, China; 2 France-China Bio-Mineralization and Nano-Structures Laboratory, Chinese Academy of Sciences, Beijing, China; 3 Department of Integrative Biology and Physiology, University of California Los Angeles, Los Angeles, CA, United States of America; 4 National Zoological Museum, Institute of Zoology, Chinese Academy of Sciences, Beijing, China; 5 University of Chinese Academy of Sciences, Beijing, China; National Research Council, ITALY

## Abstract

How animals, including mammals, can respond to and utilize the direction and intensity of the Earth’s magnetic field for orientation and navigation is contentious. In this study, we experimentally tested whether the Chinese Noctule, *Nyctalus plancyi* (Vespertilionidae) can sense magnetic field strengths that were even lower than those of the present-day geomagnetic field. Such field strengths occurred during geomagnetic excursions or polarity reversals and thus may have played an important role in the evolution of a magnetic sense. We found that in a present-day local geomagnetic field, the bats showed a clear preference for positioning themselves at the magnetic north. As the field intensity decreased to only 1/5^th^ of the natural intensity (i.e., 10 μT; the lowest field strength tested here), the bats still responded by positioning themselves at the magnetic north. When the field polarity was artificially reversed, the bats still preferred the new magnetic north, even at the lowest field strength tested (10 μT), despite the fact that the artificial field orientation was opposite to the natural geomagnetic field (*P*<0.05). Hence, *N*. *plancyi* is able to detect the direction of a magnetic field even at 1/5th of the present-day field strength. This high sensitivity to magnetic fields may explain how magnetic orientation could have evolved in bats even as the Earth’s magnetic field strength varied and the polarity reversed tens of times over the past fifty million years.

## Introduction

Many animals rely on the Earth’s magnetic field for spatial orientation and navigation, especially migratory species of birds [1, 2] and pigeons [3], fish [4–6], insects [7, 8], sea turtles [9, 10], lobsters [11, 12], and bats [13, 14]. Nevertheless, it is poorly understood how they respond to changes of the geomagnetic field when the magnetic field strength is much weaker than at present-day, for examples, during geomagnetic polarity reversals (the positions of magnetic north and magnetic south are interchanged) or geomagnetic excursion (a short-lived variation colatitude of the virtual geomagnetic pole is greater than 45 degrees from previous position). The intensities of the present-day geomagnetic field vary between 23 μT at the equator and 66 μT at polar regions [15]. Paleomagnetic studies have shown that dipolar field intensity may drop to 10% during geomagnetic polarity reversals or geomagnetic excursions [16–20]. Hence, such dramatic changes of the strength and direction of the geomagnetic field may pose a significant challenge for the evolution of any magnetic orientation in organisms.

Recently, Winklhofer and co-workers found that the avian magnetic compass could be tuned to respond to very low magnetic intensities, even down to 4 μT, indicating the considerable plasticity of the avian magnetic compass [21]. It is unknown, however, if mammals can also detect such weak magnetic fields. Bats are the second most abundant order of mammals and possess excellent flying skills. Similar to birds, many bats also migrate over large distances, crossing over a wide range of latitudes where geomagnetic field strengths and directions vary. Since bats originated in Laurasia in the early Eocene (52–50 Mya) [22], bats have experienced tens to hundreds of geomagnetic polarity reversals and geomagnetic excursions [23–25]. Since the last Brunhes-Matuyama reversal at 780 ka B.P., there were more than ten geomagnetic excursions [26]. Hence the question emerges if bats (and other mammals) could have responded to the changes of the geomagnetic field in the past (and if they will be able to do so in the future).

A pioneering study on magnetoreception in bats [13] found that big brown bats (*Eptesicus fuscus*) used the Earth’s magnetic field as compass in displacement studies. An independent laboratory investigation demonstrated that the Chinese noctule (*Nyctalus plancyi*), responded to the polarity of the magnetic field while roosting [14]. Recent works revealed that bats could calibrate their magnetic compass using polarization cues at sunset [27, 28]. These behavioral data strongly suggest that bats use magnetic compass for orientation (during migration as well as while roosting). Very limited data suggest that bats may have specialized sensory cells that contain freely rotating magnetite (Fe_3_O_4_) particles, which may serve as magnetic sensors in bat head [29, 30].

It is still unclear, however, whether bats can sense directional changes and weak field strengths that were associated with either geomagnetic polarity reversals or geomagnetic excursions. Therefore, in this study we attempted to tackle the enigmatic question if a bat can sense weak geomagnetic fields. The Chinese noctule, *Nyctalus plancyi*, which is endemic to China, is a fast aerial hawking bat that migrates to its breeding sites in March from unknown winter roost each year [31, 32]. Our previous work [14] has demonstrated that *N*. *plancyi* uses a polarity compass for orientation during roosting: the bats preferred to roost at the magnetic north when exposed to an artificial magnetic field with twice the strength of the local geomagnetic field (100 μT). As the magnetic north was reversed but field strength did not change, the bats always followed the position of the altered magnetic north.

The present study extends this previous work by examining the ability of *N*. *plancyi* to detect magnetic fields with very low intensities. Such high sensitivity would explain how bats could have acquired and retained their magnetic sense during evolution when such low magnetic field strengths and polarity reversals occurred numerous times. We found that the bats positioned themselves at the magnetic north at field strengths that were only 1/5^th^ of the natural geomagnetic field, and did so even when the polarity of the artificial field was reversed. The conditions tested here are comparable to both present-day and ancient geomagnetic field features. We briefly also discuss possible mechanisms underlying magnetoreception in bats.

## Materials and Methods

The experiments were performed in the Biogeomagnetic Laboratory at the Institute of Geology and Geophysics, Chinese Academy of Sciences, in Beijing from March to October 2013, avoiding time periods during which the bats normally undergo torpor and hibernation. The bat species used in this study was the *N*. *plancyi*, which were collected from the Dule Temple in Tianjin (40°5' N, 117°4' E) at the end of September of 2012 using mist nets. After the completion of the experiments, all individuals were returned to their site of origin and released.

### Ethical statement

The collection and experiment of the bats was approved by the Chinese Academy of Sciences and the administration of Dule Temple. The Ethics Committee of the Chinese Academy of Sciences on Vertebrate Animals Experiments and the Institute of Geology and Geophysics Administrative Panel on Laboratory Animal Care approved all experimental procedures.

### Magnetic field conditions

Bats were maintained in a basement laboratory at constant temperature and relative humidity (22±1°C; 60±1%; temperature and humidity meter THG312, Oregon Scientific, Portland, Oregon) throughout the course of the experiments. Because the *N*. *plancyi* are highly social animals, we used a group of six individuals (all males) in all conditions tested (Logistical reasons limited us to a sample size of six bats). The mean body length of the bats was 8.6±0.4 cm and the mean weight 31.5±1.7 g. The weight of the bats was monitored daily to ensure that weight loss never exceeded 10% of the original weight. The group of six bats was placed into a round plastic basket (30 cm inner diameter, 30 cm inner height) with small holes in its sides. The holes provided for ventilation of the inside with fresh air and also allowed the bats to freely move around and hang from the uppermost sides of the basket. The top of the basket was covered with a transparent glass lid, allowing for continuous infrared camera monitoring of the bats (S1 Fig).

The schematic diagram of this experimental apparatus followed the one described in [14]. The round plastic basket was placed in the center of a homogeneous magnetic field, which was generated by a custom-made tri-axial square Helmholtz coil system [33, 34]. The coil system is composed of three pairs of orthogonally aligned square coils (D = 1.5 m). Each pair of coils is independently controlled by a DC power supply (DH1715-3, DaHua Electronic Corporation, Beijing, China), generating a magnetic field in one specific axis—X, Y or Z (the number of wire loops is 190, 114, and 228, respectively). This coil system provided a uniform net magnetic field in the coil center space no less than 45×45×45 cm. The coil system was covered with thick black fabric to block out any external light. The power supplies of coils were placed in a neighboring room to keep away any noise. The experimental setup was free of any human disturbance, ambient noise, or natural light (no acoustic or visual cues provided during the entire experiments). Roosting positions of the bats were automatically recorded via an infrared camera (DS-2CC502P-IPT, Hikvision Digital Technology Corporation, Hangzhou, China). Before each magnetic condition started, the stability and homogeneity of the designed magnetic fields produced at the position of the experimental basket were monitored using an APS 520 3-axis fluxgate magnetometer (Applied Physics Systems, Mountain View, California) for 3 consecutive days. Details of used magnetic fields are present in S1 Table.

### Experimental procedures

The roosting behavior of the bats was recorded for 10 consecutive days for each magnetic field condition. During the experimental period, the bats were daily removed from the experimental basket at 6:40 p.m. every day for a period of 30 min and transferred into a holding cage where they were provided with food (mealworms) and water. Meanwhile, the experimental basket was thoroughly cleaned using water followed by 75% alcohol to eliminate any odor cues and then returned to the coil system. After feeding, the bats were returned to the center at the bottom of the cleaned experimental basket, starting an orientation record anew. Between each magnetic field condition run, the bats were allowed to recover for 7 days in the local normal geomagnetic field in order to eliminate any potential disturbance from the last tested magnetic field condition.

We initially tested the roosting behavior of the bats under local normal geomagnetic field condition (51 μT). Then, we tested the reversed polarity condition using the same strength (51 μT). Next, we pseudorandomly examined the roosting position of the bats responding to low field strengths, i.e., value of 1/3^rd^ (17 μT), 1/4^th^ (13 μT), or 1/5^th^ (10 μT) of the local geomagnetic field and also to a polarity-reversed field at the weakest intensity (10 μT).

In this study, we repeated the exposure to 1/5^th^ (10 μT) of the geomagnetic field twice and a polarity-reversed field at this low intensity. We tested the bats in a natural geomagnetic field experiment once more serving as additional control.

### Statistics

All statistical analysis was conducted in MATLAB (Mathworks Inc.). We used a MATLAB toolbox for Circular Statistics to analyze the data on the bats’ roosting positions [35]. The upper rim of the basket was divided into 72 equal sectors marked by holes, which allowed us to determine the angle of the bats' roosting orientation with 5 degrees accuracy. The magnetic north in the natural geomagnetic field was defined as zero degree, marked by the letter “N” on the rim of the basket. In all trials, the position of the basket did not change, including during the polarity reversals.

Due to their highly social nature, the bats usually clustered together when roosting. The angular value of their roosting position was obtained by determining the angle at the center of the cluster. If the bats happened to form more than 1 cluster by splitting up into several (no more than 3) groups, which occurred only very rarely, the average angular value was derived by vector calculation. The data were discarded, however, if the bats were distributed over more or less the entire surface of the basket or if they were continuously moving.

The data of the roosting position of the bat cluster was collected at 30 min intervals during the experimental time. The daily session was ended by 30 min break for feeding and watering, since the tested bats were removed from the experimental field, exposed to natural magnetic field for 30 min, and the bats had to re-determine the orientation of the magnetic field after being returned to the tested magnetic field condition (see Methods above). Therefore, we considered the daily data set obtained for each magnetic field condition as 10 independent repetitions.

We determined the distribution of the bats in the basket as circular means, which were expressed as vector mean values, not the classic arithmetic mean value [35]. Vector analysis then yielded the mean direction of the entire group of bats for each magnetic field condition for each 10-day testing period. The length of the vector mean indicated how concentrated the data sample was around the mean direction (max. value is 1; for details, see r_b_ values in Table 1, and S3 Table). We illustrated the distribution of the different angular orientation (12 angle bins) graphically in a rose (theta) diagram, covering 30 degrees each. The results for replicated trials at the lowest intensity 1/5^th^ of the natural geomagnetic field strength are shown in S2 Fig. A nonparametric Rayleigh test and a Watson U2 test assessed if the orientation was significantly different from a random distribution (see p values in Table 1 and S3 Table) and compared the mean directions of the bat clusters between different magnetic field conditions. The daily orientation data for each magnetic condition were also assessed by a nonparametric Rayleigh test (p values not shown).

**Table 1:** Summary of the mean directions of the bat cluster and results of Rayleigh test for a preferred orientation in different magnetic fields.

Field conditions	No. data	a_b_ (°)	r_b_	p
GMF	394	25	0.53	<0.001
1/3^rd^ GMF	451	21	0.50	<0.001
1/4^th^ GMF	433	25	0.62	<0.001
1/5^th^ GMF	466	27	0.47	<0.001
Reversed_GMF	454	162	0.52	<0.001
Reversed_1/5^th^ GMF	462	158	0.56	<0.001

GMF: natural geomagnetic field strength.

a_b_: the mean direction

r_b_: the magnitude of the mean resultant vector

p: a significant level. p<0.05 indicating a preferred direction; p>0.05 indicating random directions.

## Results

**Fig 1:**
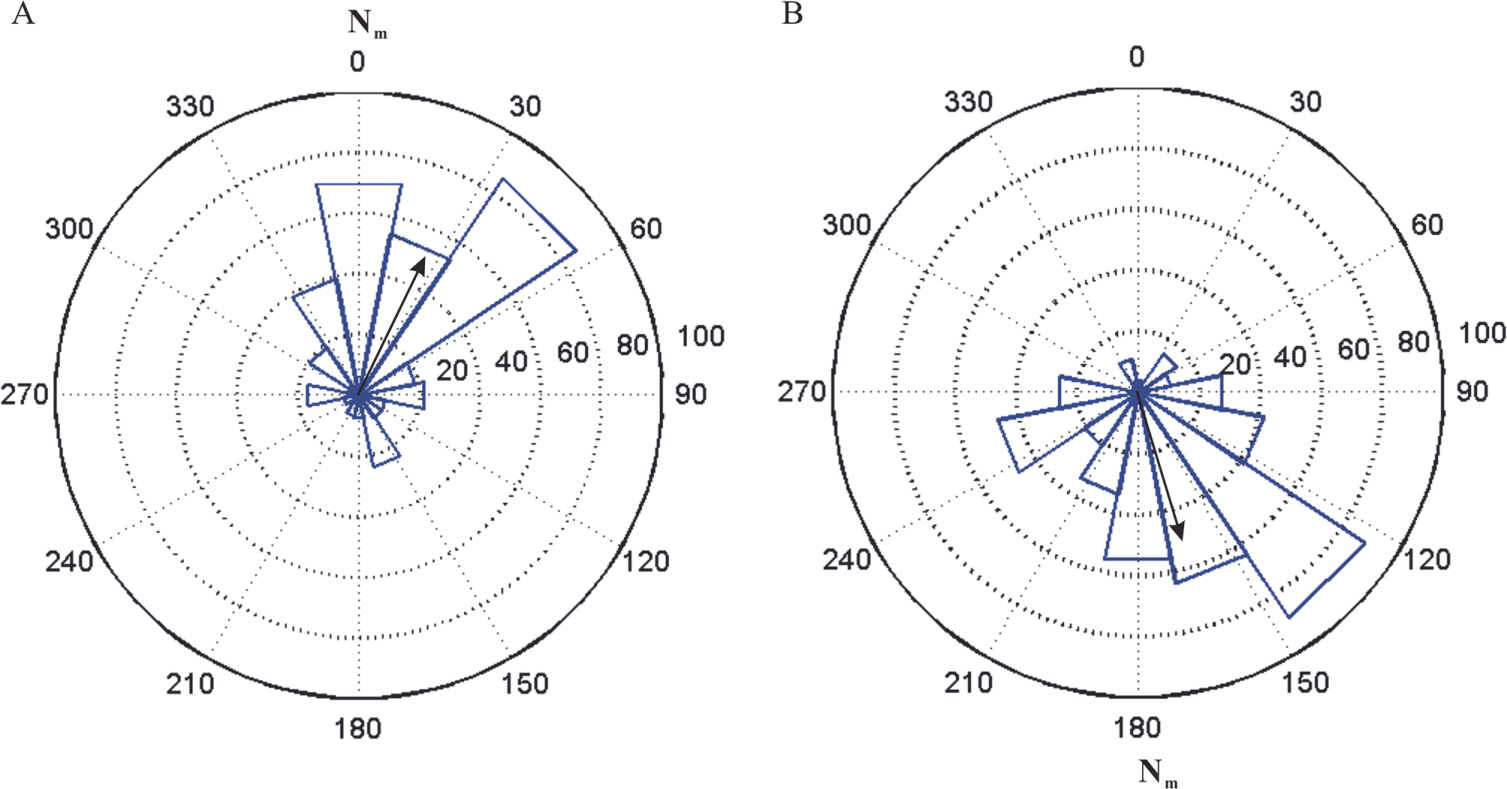
Angular histograms of roosting locations in the natural geomagnetic field (A) and a polarity reversed magnetic field (B). Field intensity is 51μT. Black arrows indicate the direction and magnitude of the mean resultant vector. N_m_: magnetic north.

Fig 1 shows that *N*. *pancyi* in a magnetic field with natural strength preferred to roost near the magnetic north (0°). The mean direction in the natural geomagnetic field was 25°. When the polarity was reversed, the bats changed their roosting preference to the geographic south (162°), i.e. they remained at the (now new, artificial) magnetic north. The mean direction of the bats in both the natural geomagnetic field and the polarity-reversed field were significantly different from a random distribution and from each other (p<0.05) (Table 1).

**Fig 2:**
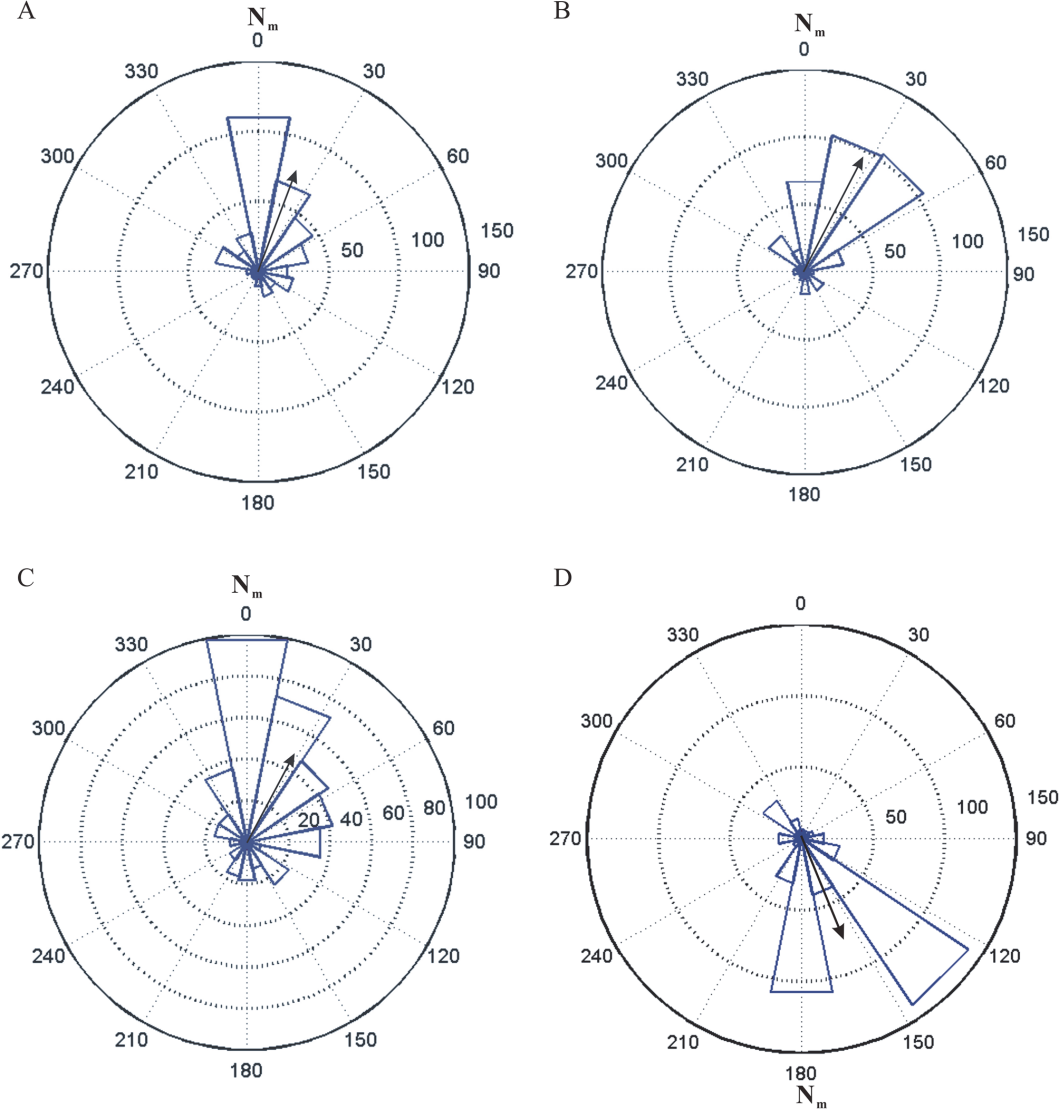
Angular histograms of roosting locations in different magnetic field strengths and in reversed polarity at the weakest field strength. (A) 1/3^rd^ of natural field strength. (B) 1/4^th^ of natural field strength. (C) 1/5^th^ of natural field strength. (D) reversed polarity of a magnetic field with 1/5^th^ of natural field strength. N_m_: magnetic north.

Fig 2 demonstrates that the bats statistically significantly preferred to roost at the magnetic north at 1/3^rd^, 1/4^th^, and 1/5^th^ of the strength of the natural geomagnetic field. The lengths of the mean vectors, r_b_, were all >0.5, except for the case of the weakest field (Table 1). Under all magnetic field conditions, from natural down to 1/5^th^ and normal to reversed polarities, the tested bat’s roosted near the magnetic north. Although the direction data for the weakest intensity (1/5^th^ of the natural strength) appeared slightly more scattered than those for 1/3^rd^ and 1/4^th^ of the natural strength (Fig 2A–2C), statistical analysis indicated that the mean directions under all three weak magnetic field conditions were not significantly different (p>0.05). When we reversed the polarity of the weakest field (Fig 2D), the mean direction of the bat cluster is 158°, indicating that the bats still preferred the (new, artificial) magnetic north at a statistically significant level.

This is further confirmed by repeat tests in the weakest field strength (10 μT) for normal polarity and reversed polarity (S2 Fig). Their a and r values are present in S3 Table.

All magnetic field conditions yielded results that were significantly different from random roosting behavior (Table 1 and S3 Table). All daily orientation data of the bats were also significantly different from a random distribution (p<0.05, p values not shown in S2 and S4 Tables). Individual analysis of the daily orientation data for each magnetic condition also revealed a consistent preference for the magnetic north, only a few data deviating from the magnetic north. It is noteworthy, however, that the a_b_ and r_b_ values of the daily orientation data indicated that the bats did not remain at the exact same position throughout the entire day. Instead, they often moved around slightly, nevertheless always staying close to the magnetic north. These results clearly indicate a remarkable sensitivity of *N*. *plancyi* to magnetic field strengths of only 10 μT, which is only 1/5^th^ of the present-day local geomagnetic field value.

Although generally all bats eventually congregated at the magnetic north at even the weakest magnetic field strength tested, the time period that passed until the bats did so (the “response time”) depended on the magnetic field strength. At field strength greater than 1/5^th^ of the natural strength, the bats began roosting near the magnetic north more quickly than at the lowest intensity. At a field strength of 1/5^th^ of the natural strength (either at the normal or the reversed polarity), the bats roosted at random positions on the rim of the basket (S1D Fig) for the first three experimental days, which did not allow us to determine preferred roosting angle. After that, the bats began to exhibit a clear preference for a particular orientation. Hence, we began data collection on the 4^th^ exposure day and continued for the subsequent 10 days.

Interestingly, we noted that every time either the field strength was changed and/or the field polarity was reversed, the bats also reduced their food intake for the first 1–3 daily exposures following the changes. Only after this initial period, the bats appeared to habituate to the changed magnetic condition and their food intake returned to normal.

## Discussion

The results of the present study demonstrate a remarkable sensitivity of a migratory vespertilionid bat, the Chinese noctule (*N*. *plancyi*), to magnetic fields as weak as 1/5^th^ of the natural geomagnetic field strength, i.e. 10 μT. This sensitivity was evident in responses to the normal orientation of fields with different strength but also to a polarity-reversed field of even the weakest field strength. Under all tested magnetic field conditions, the bats always roosted near the magnetic north. This further corroborates the finding of polarity-based compass of *N*. *plancyi* in our earlier study [14]. It remains unclear, however, what the benefits of roosting near the magnetic north might be.

This high sensitivity of the magnetic orientation in bats, the first of its kind in mammals, is comparable to that recently observed in birds [21]. This ability of bats and birds to detect even very weak magnetic fields may have important implications in how these animals cope with the significant geomagnetic field changes that occurred over their course of evolution. Based on the data presented here and our earlier study [14], *N*. *plancyi* is able to detect field strengths ranging from 10 to 100 μT. This allows them to orient themselves across the entire range of present-day global geomagnetic field strengths. The remarkable sensitivity to magnetic field strengths of only 10 μT indicates that these bats may have been able to orientate themselves even in the low field strengths that occurred during geomagnetic polarity reversal or excursion. It may enable bats to use the Earth's magnetic field coordinate for orientation and navigation, similar to that of sea turtles, lobsters, or pigeons [10, 11, 36].

Interestingly, numerous other mammals, such as cows, red foxes, or dogs, also appear to be able to sense magnetic fields and align their bodies along the magnetic north-south axis when they rest, hunt or defecate. The underlying mechanism and the purpose of this behavior remain unknown, however [37–40]. Finally, the prolonged delay in the response to the weakest magnetic field tested here (1/5^th^ of the natural field strength, or 10 μT) suggests that this field strength may be close to the threshold of magnetoreception for these bats. Due to the technical difficulty to generate very stable magnetic fields with even lower-intensity, we refrained here from testing magnetic field strengths below 10 μT.

The physiological mechanisms underlying magnetoreception in bats and other animals are poorly understood. The most widely accepted hypotheses include photoreceptor-based and/or magnetite-based magnetoreception [41–43]. Because the location of any magnetite magnetoreceptor in bats is currently still unknown, we could not determine the potential role of magnetite particles in magnetic orientation, for example by pharmacologically blocking sites containing such magnetite particles, as performed in birds [21]. Nevertheless, the bats used here naturally roost in dark locations, such as in caves, during the day and only in the darkness of night emerge from their roost sites to forage. Hence it appears unlikely that a light-based mechanism of magnetoreception is at work here.

Several lines of evidence rather suggest a magnetite-based mechanism: First, magnetite-based magnetoreceptors should be able to detect both, direction and intensity of a magnetic field [44, 45]. We recently measured magnetic properties of the brain tissues of migratory as well as non-migratory bats using a “superconducting quantum interference device” (SQUID) magnetometer. Interestingly, the isothermal remanent magnetization acquisition results indicated that the amount of soft magnetic particles (magnetites) was higher in migratory than in non-migratory species, implying a magnetite-based reception mechanism [30]. Furthermore, Holland and co-workers used pulse-remagnetization experiments detecting primarily iron minerals indicating that the body of bats contains freely rotating magnetite particles [29]. Such a magnetite-based magnetoreception has also been suggested for different mole-rat species [46, 47].

In summary, our results hint at the possibility that migratory bats may have the potential to detect magnetic fields and potentially use them for orientation and maybe even navigation. We found for the first time that the magnetic sense in the Chinese noctule, *N*. *plancyi*, could even detect extremely weak magnetic field (10 μT). This could explain how magnetoreception was able to evolve over a period of time when the Earth’s magnetic field underwent numerous and dramatic weakenings of its field strength during geomagnetic reversals or excursions. However, the location of any magnetoreceptors, which are most likely magnetite-based, in mammals including bats and how they function remains unclear. Further histological and physiological studies may aid in determining where such magnetoreceptors are found and how they work.

## Supporting Information

S1 Fig Snapshots of the bat cluster in the experimental chamber while roosting in different magnetic fields. (TIF)

S2 Fig Angular histograms of roosting locations in replicated trials of the weakest field strengths (1/5^th^ GMF, 10μT) and of reversed polarity at the weakest field strength. (TIF)

S1 Table Details of the magnetic field conditions tested. (DOC)

S2 Table Daily vector averages of the bat cluster for exposures to six different magnetic field conditions. (DOC)

S3 Table Summary of the mean directions of the roosting location of the bat cluster (a_b_ in degrees) and results of Rayleigh test for a preferred orientation in replicated trials of lowest intensity magnetic fields (r_b_, p). (DOC)

S4 Table Daily vector averages for the bat cluster when exposed to the lowest magnetic field intensity. Data cover one complete exposure series (10 days).
(DOC)
