# Supplementary figures and images for: Bats Respond to Very Weak Magnetic Fields
Source: PLoS One. 2015 Apr 29;10(4):e0123205. doi: 10.1371/journal.pone.0123205 (PMC4414586; doi:10.1371/journal.pone.0123205)

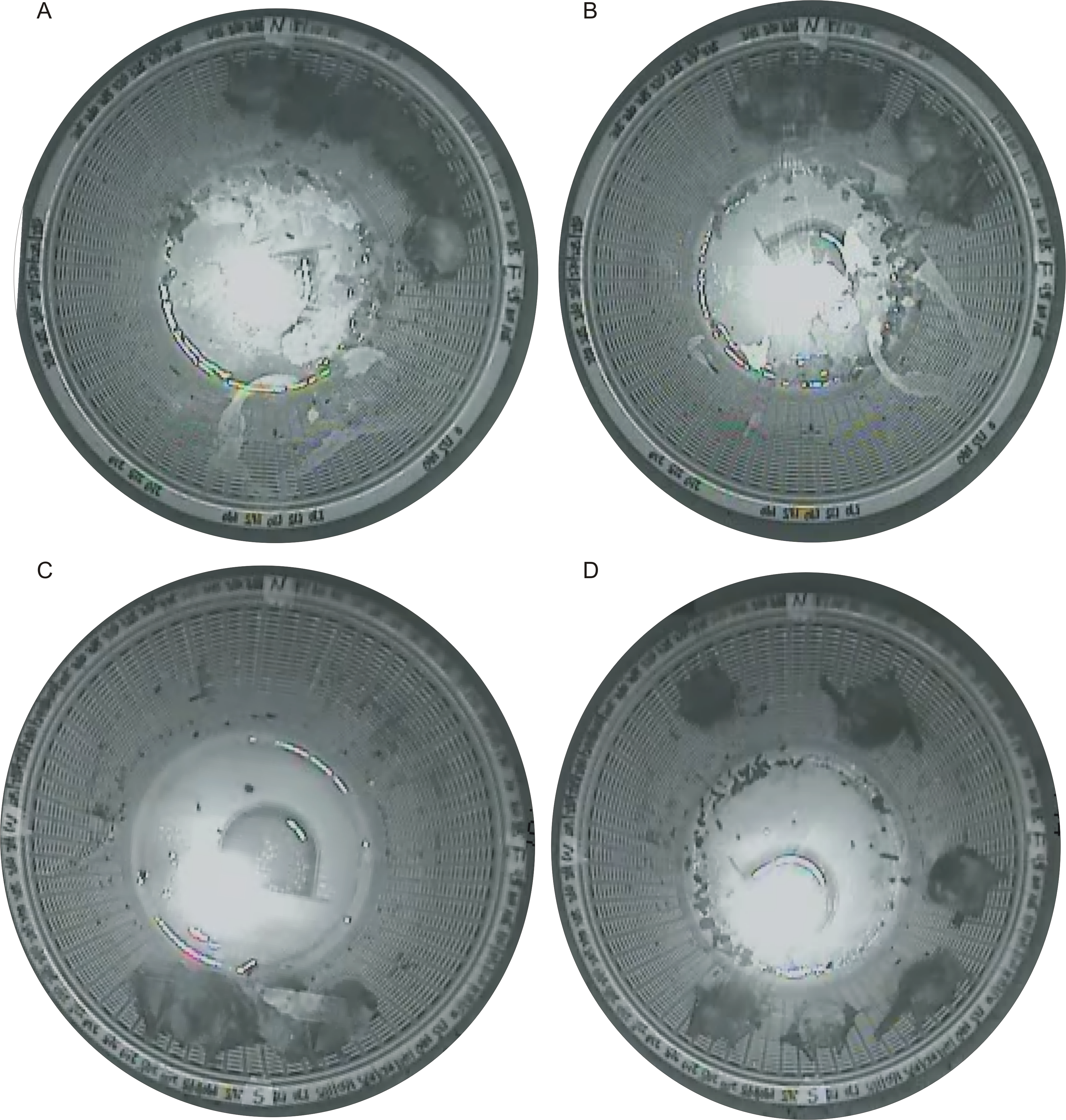

Supplement: S1 Fig — (TIF) [file pone.0123205.s001.tif]

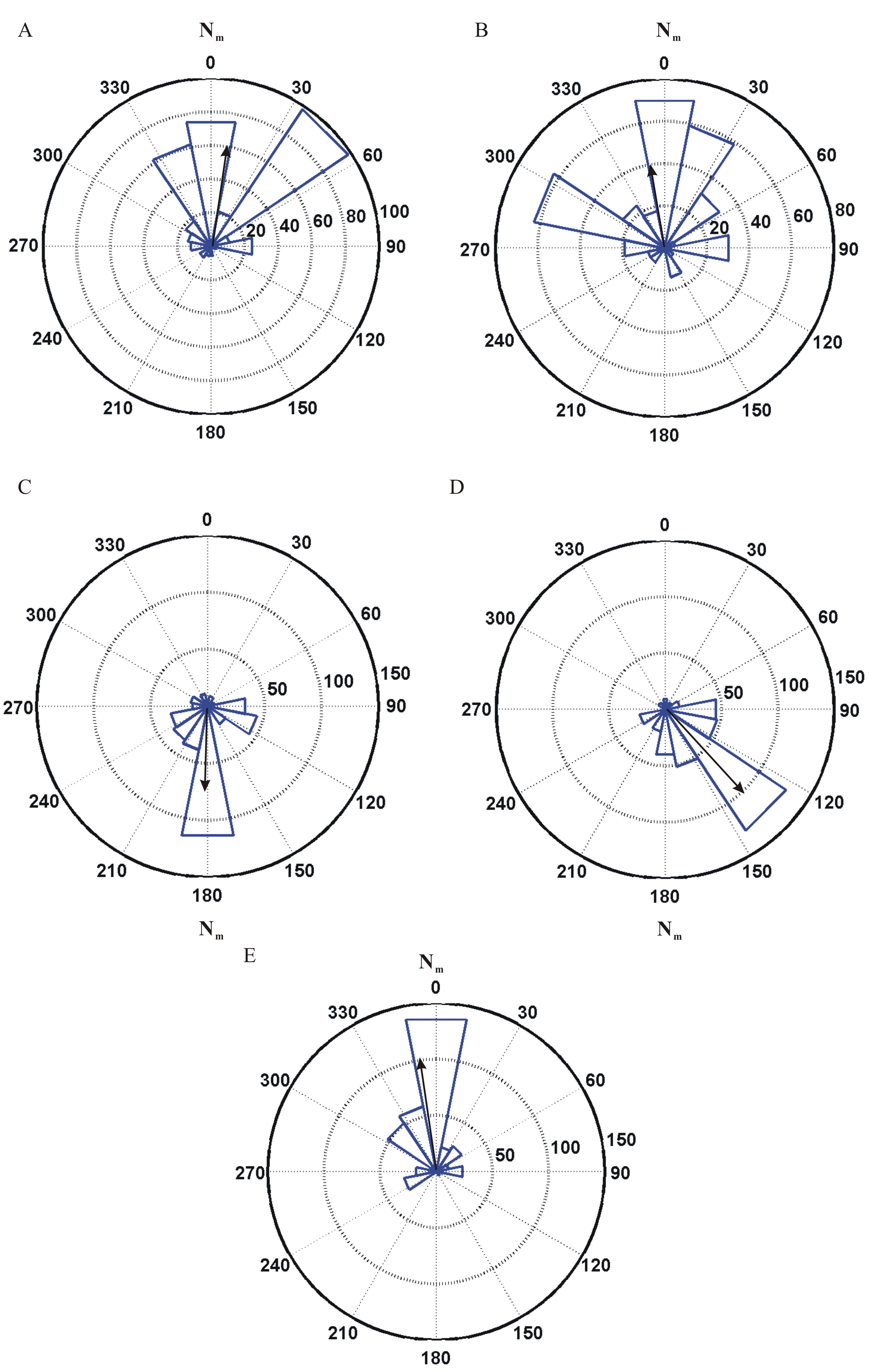

Supplement: S2 Fig — (TIF) [file pone.0123205.s002.tif]
