# Supplementary material for: Bats Respond to Very Weak Magnetic Fields
Source: PLoS One. 2015 Apr 29;10(4):e0123205. doi: 10.1371/journal.pone.0123205 (PMC4414586; doi:10.1371/journal.pone.0123205)
Supplement: S1 Table — (DOC) [file pone.0123205.s003.doc]

**S1 Table. Details of the magnetic field conditions tested.**

| Magnetic field | Local geomagnetic field (GMF) | 1/3^rd^ GMF | 1/4^th^ GMF | 1/5^th^ GMF | Reversed, GMF | Reversed, 1/5^th^ GMF |
| --- | --- | --- | --- | --- | --- | --- |
| Intensity (µT) (mean±S.D) | 51.3±0.3 | 17.0±0.3 | 13.2±0.3 | 10.6±0.5 | 49.4±1.6 | 10.1±0.8 |
| Declination (°) | 1.2±3.8 | 2.7±3.7 | 4.7±3.4 | 5.4±4.7 | 185.2±2.9 | 178.4±5.8 |
| Inclination (°) | 60.6±0.2 | 60.9±2.4 | 61.7±1.8 | 62.9±4.2 | 60±0.5 | 58.6±2.5 |
