# Supplementary material for: Bats Respond to Very Weak Magnetic Fields
Source: PLoS One. 2015 Apr 29;10(4):e0123205. doi: 10.1371/journal.pone.0123205 (PMC4414586; doi:10.1371/journal.pone.0123205)
Supplement: S2 Table — (DOC) [file pone.0123205.s004.doc]

**S2 Table. Daily vector averages of the bat cluster for exposures to six different magnetic field conditions.**

| Tested day | GMF | | 1/3^rd^ GMF | | 1/4^th^ GMF | | 1/5^th^ GMF | | Reversed, GMF | | Reversed, 1/5^th^ GMF | |
| --- | --- | --- | --- | --- | --- | --- | --- | --- | --- | --- | --- | --- |
|  | a_b_ , r_b_ | | a_b_ , r_b_ | | a_b_ , r_b_ | | a_b_ , r_b_ | | a_b_ , r_b_ | | a_b_ , r_b_ | |
| 1st | 49° | 0.99 | 154° | 0.53 | 81° | 0.28 | 140° | 0.65 | 149° | 0.97 | 177° | 0.99 |
| 2nd | 34° | 0.84 | 118° | 0.68 | 39° | 0.86 | 54° | 0.78 | 196° | 0.63 | 141° | 0.95 |
| 3rd | 28° | 0.94 | 3° | 0.89 | 31° | 0.88 | 166° | 0.49 | 247° | 0.96 | 125° | 0.95 |
| 4th | 44° | 0.98 | 344° | 0.78 | 184° | 0.5 | 36° | 0.36 | 224° | 0.70 | 160° | 0.83 |
| 5th | 310° | 0.75 | 67° | 0.8 | 5.4° | 0.92 | 51° | 0.84 | 167° | 0.95 | 185° | 0.94 |
| 6th | 7° | 0.79 | 14° | 0.93 | 32° | 0.99 | 353° | 0.82 | 85° | 0.48 | 322° | 0.59 |
| 7th | 54° | 0.88 | 29° | 0.89 | 326° | 0.83 | 350° | 0.83 | 146° | 0.87 | 323° | 0.47 |
| 8th | 63° | 0.4 | 17° | 0.81 | 5° | 0.88 | 9° | 0.97 | 111° | 0.9 | 161° | 0.82 |
| 9th | 162° | 0.69 | 303° | 0.68 | 44° | 0.89 | 15° | 0.76 | 76° | 0.35 | 123° | 0.83 |
| 10th | 344° | 0.9 | 359° | 0.8 | 41° | 0.94 | 0° | 0.46 | 162° | 0.66 | 142° | 0.57 |

GMF, geomagnetic field; a_b_: mean direction, r_b_: magnitude of the mean resultant vector.
