# Supplementary material for: Bats Respond to Very Weak Magnetic Fields
Source: PLoS One. 2015 Apr 29;10(4):e0123205. doi: 10.1371/journal.pone.0123205 (PMC4414586; doi:10.1371/journal.pone.0123205)
Supplement: S3 Table — (DOC) [file pone.0123205.s005.doc]

**S3 Table. Summary of the mean directions of the roosting location of the bat cluster (a_b_ in degrees) and results of Rayleigh test for a preferred orientation in replicated trials of lowest intensity magnetic fields (r_b_, p).**

| Field conditions | No. data | a_b_ (°) | r_b_ | p |
| --- | --- | --- | --- | --- |
| GMF(2^nd^) | 392 | 350 | 0.67 | <0.001 |
| 1/5^th^ GMF(2^nd^) | 362 | 10 | 0.63 | <0.001 |
| 1/5^th^ GMF(3^rd^) | 361 | 353 | 0.49 | <0.001 |
| Reversed, 1/5^th^ GMF (2^nd^) | 385 | 185 | 0.51 | <0.001 |
| Reversed, 1/5^th^ GMF (3^rd^) | 399 | 137 | 0.68 | <0.001 |

GMF: natural geomagnetic field strength. No. data, the number of collected data; a_b_, mean direction; r_b_, magnitude of the mean resultant vector; p, a significant level. p<0.05 indicating a preferred direction; p>0.05 indicating random directions.
