# Supplementary material for: Bats Respond to Very Weak Magnetic Fields
Source: PLoS One. 2015 Apr 29;10(4):e0123205. doi: 10.1371/journal.pone.0123205 (PMC4414586; doi:10.1371/journal.pone.0123205)
Supplement: S4 Table — Data cover one complete exposure series (10 days). (DOC) [file pone.0123205.s006.doc]

**S4 Table. Daily vector averages for the bat cluster when exposed to the lowest magnetic field intensity. Data cover one complete exposure series (10 days).**

| Test day | GMF(2^nd^) | | 1/5^th^ GMF(2^nd^) | | 1/5^th^ GMF(3^rd^) | | Reversed, 1/5^th^ GMF (2^nd^) | | Reversed, 1/5^th^ GMF (3^rd^) | | |  |
| --- | --- | --- | --- | --- | --- | --- | --- | --- | --- | --- | --- | --- |
|  | a_b_ , r_b_ | | a_b_ , r_b_ | | a_b_ , r_b_ | | a_b_ , r_b_ | | a_b_ , r_b_ | | |  |
| 1st | 44° | 0.69 | 191° | 0.46 | 102° | 0.39 | 179° | 0.5 | 135° | 0.96 |  | |
| 2nd | 36° | 0.88 | 17° | 0.82 | 6° | 0.7 | 190° | 0.99 | 269° | 0.49 |  | |
| 3rd | 337° | 0.92 | 346° | 0.85 | 1° | 0.92 | 101° | 0.9 | 133° | 0.95 |  | |
| 4th | 330° | 0.77 | 330° | 0.9 | 2° | 0.66 | 264° | 0.7 | 146° | 0.8 |  | |
| 5th | 319° | 0.81 | 83° | 0.64 | 51° | 0.49 | 233° | 0.6 | 139° | 0.6 |  | |
| 6th | 332° | 0.91 | 45° | 0.74 | 8° | 0.85 | 251° | 0.77 | 153° | 0.45 |  | |
| 7th | 358° | 0.95 | 344° | 0.82 | 303° | 0.51 | 201° | 0.98 | 125° | 0.86 |  | |
| 8th | 32° | 0.83 | 30° | 0.74 | 307° | 0.62 | 190° | 0.67 | 127° | 0.88 |  | |
| 9th | 344° | 0.64 | 11° | 0.81 | 271° | 0.47 | 181° | 0.86 | 128° | 0.92 |  | |
| 10th | 298° | 0.42 | 12° | 0.84 | 24° | 0.69 | 113° | 0.97 | - | - |  | |
|  | | | | | | | | | | |  | |

GMF, geomagnetic field; a_b_: mean direction; r_b_: magnitude of the mean resultant vector; “-”, no data.
